# The emergence of *Vibrio* pathogens in Europe: ecology, evolution, and pathogenesis (Paris, 11–12th March 2015)

**DOI:** 10.3389/fmicb.2015.00830

**Published:** 2015-08-13

**Authors:** Frédérique Le Roux, K. Mathias Wegner, Craig Baker-Austin, Luigi Vezzulli, Carlos R. Osorio, Carmen Amaro, Jennifer M. Ritchie, Tom Defoirdt, Delphine Destoumieux-Garzón, Melanie Blokesch, Didier Mazel, Annick Jacq, Felipe Cava, Lone Gram, Carolin C. Wendling, Eckhard Strauch, Alexander Kirschner, Stephan Huehn

**Affiliations:** ^1^Unié Physiologie Fonctionnelle des Organismes Marins, Ifremer, Plouzané, France; ^2^CNRS, UMR 8227, Integrative Biology of Marine Models, Station Biologique de Roscoff, Sorbonne Universités, UPMC Paris 06, Roscoff cedex, France; ^3^Coastal Ecology, Alfred Wegener Institute Helmholtz Centre for Polar and Marine Research, List, Germany; ^4^Cefas, Weymouth, UK; ^5^Department of Earth, Environmental and Life Sciences, University of Genoa, Genoa, Italy; ^6^Departamento de Microbioloxía e Parasitoloxía, Instituto de Acuicultura, Universidade de Santiago de Compostela, Santiago de Compostela, Spain; ^7^Estructura de Investigación Interdisciplinar en Biotecnología y Biomedicina, Department of Microbiology and Ecology, University of Valencia, Valencia, Spain; ^8^Faculty of Health and Medical Sciences, University of Surrey, Guildford, UK; ^9^UGent Aquaculture R&D Consortium, Ghent University, Ghent, Belgium; ^10^Interactions Hôtes-Pathogènes-Environnements, UMR 5244, CNRS, Ifremer, Université de Perpignan Via Domita, Université de Montpellier, Montpellier, France; ^11^Laboratory of Molecular Microbiology, Global Health Institute, School of Life Sciences, Ecole Polytechnique Fédérale de Lausanne, Lausanne, Switzerland; ^12^Département Génomes et Génétique, CNRS UMR3525, Unité Plasticité du Génome Bactérien, Institut Pasteur, Paris, France; ^13^Institute for Integrative Biology of the Cell, CEA, CNRS, Université Paris-Sud, Orsay, France; ^14^Laboratory for Molecular Infection Medicine Sweden, Department of Molecular Biology, Umeå Centre for Microbial Research, Umeå University, Umeå, Sweden; ^15^Department of Systems Biology, Technical University of Denmark, Kongens Lyngby, Denmark; ^16^Geomar, Helmholtz Centre for Ocean Research Kiel, Kiel, Germany; ^17^Federal Institute for Risk Assessment, National Reference Laboratory for Monitoring Bacteriological Contamination of Bivalve Molluscs, Berlin, Germany; ^18^Institute for Hygiene and Applied Immunology, Medical University of Vienna, Vienna, Austria; ^19^Institute of Food Hygiene, Free University Berlin, Berlin, Germany

**Keywords:** global warming, human health, aquaculture, interactions, animal model, bacterial disease, genome plasticity, european network

## Abstract

Global change has caused a worldwide increase in reports of Vibrio-associated diseases with ecosystem-wide impacts on humans and marine animals. In Europe, higher prevalence of human infections followed regional climatic trends with outbreaks occurring during episodes of unusually warm weather. Similar patterns were also observed in *Vibrio*-associated diseases affecting marine organisms such as fish, bivalves and corals. Basic knowledge is still lacking on the ecology and evolutionary biology of these bacteria as well as on their virulence mechanisms. Current limitations in experimental systems to study infection and the lack of diagnostic tools still prevent a better understanding of *Vibrio* emergence. A major challenge is to foster cooperation between fundamental and applied research in order to investigate the consequences of pathogen emergence in natural *Vibrio* populations and answer federative questions that meet societal needs. Here we report the proceedings of the first European workshop dedicated to these specific goals of the *Vibrio* research community by connecting current knowledge to societal issues related to ocean health and food security.

## State of the Art and Perspectives of *Vibrio* Research in Europe

According to the European Environment Agency the rise of global sea surface temperature (SST) is one of the major physical impacts of climate change. However, SST in coastal European seas has increased 4–7 times faster over the past few decades than in the global oceans (Reid et al., 2011). This local increase in SST has been linked to outbreaks of *Vibrio*-associated human illness caused by *Vibrio cholerae* non O1-non-O139, *V. parahaemolyticus*, and *V. vulnificus* in several European countries (Table 1). However, the lack of mandatory notification systems for *Vibrio*-associated illnesses prevents accurate estimates of the number of *Vibrio* infections occurring in Europe. Also mass mortalities of marine animals increase in frequency (Table 1), particularly in heavily polluted coastal areas, suggesting human activities as a factor favoring disease epidemics. Prominent examples include several *Vibrio* species associated with the recent great devastation of oyster beds in France. The salmonid farming industry is constantly threatened by *V. salmonicida* and *V. anguillarum*. Moreover, different subspecies of *Photobacterium damselae* are associated with diseases in cultured fish species like sole, sea bass, sea bream and turbot, while *V. vulnificus* causes hemorrhagic septicaemia in eel, derbio, tilapia, trout and shrimps but can also cause septicemia in humans. Finally, evidence has accumulated linking *Vibrio* infections (e.g., *V. coralliilyticus*) to increasing mass mortalities of benthic corals (e.g., *Paramuricea clavata*) in the NW Mediterranean Sea.

**TABLE 1:** **Recent Vibrio-associated diseases caused by Vibrio in Europe**.

**Agent**	**Pathogenic to**	**Country**	**References**
*V. parahaemolyticus, V. vulnificus and non-O1/non-O139 V. cholerae*	Human	Germany	Huehn et al. (2014)
*V. parahaemolyticus*	Human	France	Quilici et al. (2005)
*V. parahaemolyticus*	Human	Spain	Martinez-Urtaza et al. (2004)
*V. parahaemolyticus*	Human	Italy	Ottaviani et al. (2012)
*V. cholerae*	Human	Sweden	Andersson and Ekdahl (2006)
*V. cholerae non-O1-non-O139*	Human	Italy	Ottaviani et al. (2009)
*V. cholerae non-O1-non-O139*	Human	Finland	Lukinmaa et al. (2006)
*V. cholerae non-O1-non-O139*	Human	Poland	Stypulkowska-Misiurewicz et al. (2006)
*V. cholerae non-O1-non-O139*	Human	Austria	Huhulescu et al. (2007)
*V. cholerae non-O1-non-O139*	Human	Austria	Kirschner et al. (2008)
*V. vulnificus*	Human	Denmark	Dalsgaard et al. (1996)
*V. vulnificus*	Human	Israel	Bisharat et al. (1999)
*V. vulnificus*	Human	Spain	Torres et al. (2002)
*V. vulnificus*	Human	Turkey	Partridge et al. (2009)
*V. vulnificus*	Human, finfish, crustacean	USA, Europe, Asia	Amaro et al. (2015); Oliver (2015)
*V. alginolyticus*	Human	Guernsey	Reilly et al. (2011)
*Vibrio* spp.	Human	North and Baltic Seas	Schets (2012)
*V. coralliilyticus*	Coral	Italy	Vezzulli et al. (2010b)
*V. crassostreae*	Oyster	France	Lemire et al. (2014)
*V. aestuarianus*	Oyster	France	Goudenège et al. (2014)
*Harveyi clade*	Finfish, crustaceans, mollusks	Mediterranean countries	Pujalte et al. (2003)
*V. anguillarum*	Finfish	Northern European countries	Frans et al. (2011)
*Photobacterium damselae* subsp. *Damselae*	Fish, humans, crustaceans, mollusks, cetaceans	Mediterranean countries	Rivas et al. (2013b)

To cover the large diversity of infectious vibrios, the development of operational tools to identify and detect emergent pathogens is essential to zoosanitary monitoring of cultivated species as well as on wild animal populations. Yet, compared to human pandemic strains, little is known about the virulence mechanisms of emergent environmental vibrios. This lack of knowledge may be attributed to the high genetic diversity of *Vibrio* isolates and the diversity/plurality of virulence mechanisms. To date pathogenic capacity cannot be inferred by taxonomic affiliation, because virulence factors (e.g., secretion systems, toxins) are rarely species-specific and are often shared between *Vibrio* species by lateral gene transfer. On top of that there are very few animal models to distinguish pathogenic strains and extend our understanding of the mechanisms involved in host-microbe interactions. Hence the elucidation of virulence for agent and target is a prerequisite to develop prophylactic methods to fight infectious diseases.

Due to the extent of the environmental, economical, and public health consequences resulting from *Vibrio* infections, a large scientific community is working on these bacteria in Europe. In order to join fundamental and applied research teams and to investigate the emergence of pathogens in natural *Vibrio* populations, we organized the first European workshop dedicated to the research on vibrios in Paris (11–12th March 2015), that provided a forum for experts in *Vibrio* ecology, evolution and pathogenesis to address societal issues involving ocean health and food security.

### *Vibrio* Spread in Europe linked with climate change

Vibrios preferentially grow in warm (>15°C) saline aquatic environments. Warming of marine and saline inland waters is likely to support larger numbers of *Vibrio* populations and consequently an increased risk of *Vibrio* infections. An increase in the prevalence of human infections caused by *V. parahaemolyticus*, *V. cholerae* non-O1-non-O139 and *V. vulnificus* has been recorded in Europe even at high latitudes (Baker-Austin et al., 2013). In northern Europe, the increase in reported infections corresponds both in time and space with spikes in domestically-acquired *Vibrio* cases in “heatwave” years. Similarly, samples collected in the last 60 years by the continuous plankton recorder (CPR) survey (Vezzulli et al., 2012) showed that the genus *Vibrio*, including the human pathogen *V. cholerae*, has increased in prevalence in the last 44 years in the coastal North Sea, and that this increase is correlated with warming SST. Elevated water temperatures might also facilitate the successful invasion of pathogenic variants via food trade (Nair et al., 2007), ballast water (Dobbs et al., 2013), travelers (Fillion and Mileno, 2015) or natural animals. For example, migrating birds may act as vectors of intercontinental transport of *V. cholerae* (Vezzulli et al., 2010a). The direct comparison of the population structure of *V. cholerae* from a major bird sanctuary (Lake Neusiedl, Austria), with strains collected from six other European countries revealed that several strains in the lake shared the same alleles with other European strains, consistent with pan-European transport between distant ecosystems via birds (A. Kirschner, unpublished data). As a future challenge, macro-ecological studies on the impact of climate change on *Vibrio* persistence and spread in the aquatic environment combined with studies investigating climate change effects on epidemiologically relevant variables, such as host susceptibility and exposure are needed to significantly improve prediction and mitigation strategies against the future occurrence of *Vibrio* disease outbreaks.

### Virulence as a Function of Biotic Interactions With Host and Microbiome

Virulence is a widespread phenomenon across the *Vibrio* phylogeny (Wendling et al., 2014). Its expression critically depends on biotic interactions with the host but also with other resident microbiota. On the host side, spatially-structured cross-infection experiments indicated that virulence of only distantly related *Vibrio* strains was lower when infecting oysters from the same geographic location. This suggests that oyster hosts are locally adapted and have evolved resistance to genetic factors shared within *Vibrio* populations (Wendling and Wegner, 2015). When considering interactions of *Vibrio* with the resident microbiome, the hemolymph microbiome modulates infections but is vulnerable to environmental disturbance (Lokmer and Wegner, 2015). Accordingly, *Vibrio* disease cannot be seen as an isolated event but needs to be considered in the context of the microbiome, which includes other non-virulent *Vibrio.* Indeed, the successive replacement of non-virulent with virulent strains during oyster infections occurs in the natural environment (Lemire et al., 2014) and the amplification of virulence in the presence of non-virulent strains suggests that also non-virulent strains contribute directly or indirectly to the development of disease. Future research on *Vibrio* disease should therefore focus on the higher order biotic interactions between the environment, the host and the pathogenic as well as the non-pathogenic fractions of microbial communities.

A key feature of the interaction between microbes within a community is the production of molecules that determine behavior like antagonism, competition or cooperation. Cell-to-cell communication in vibrios coordinates virulence gene expression based on the biotic and abiotic environment (Defoirdt, 2014). For example, the three-channel quorum sensing (QS) system of *V. harveyi* controls the pathogenicity of the bacterium toward different aquatic hosts (Defoirdt and Sorgeloos, 2012; Pande et al., 2013), and our most recent research revealed that another signaling molecule, indole, controls the virulence of *V. anguillarum* toward sea bass larvae (Li et al., 2014b). Another potential signaling mechanism has been described based on the production and release of high concentrations of D-amino acids into the extracellular milieu (Lam et al., 2009). First discovered in *V. cholerae*, these D-amino acids are different from those known to be part of the cell wall in bacteria (D-Ala and D-Glu) and were therefore called non-canonical D-amino acids (NCDAAs; Cava et al., 2011a). NCDAAs released into the media by producer strains can affect non-producer organisms beneficially or detrimentally in a particular niche (Cava et al., 2011b; Alvarez et al., 2014). The possible implications of NCDAAs in the biological processes of co-inhabitants still remains to be investigated but the enormous energy demand suggests that these molecules should have a great impact in poly-microbial communities. Finally several strains of *Vibrio* have been demonstrated to produce potent antibacterial agents (andrimid and holomycin) or agents that block QS regulated genes (solonamides, ngercheumicin) in human pathogens (Wietz et al., 2010; Mansson et al., 2011; Kjaerulff et al., 2013; Nielsen et al., 2014). Comparative and functional genomics using software like antiSMASH (Medema et al., 2011) could identify the genetic determinants of these secondary metabolites (polyketide synthases and non-ribosomal peptide synthetases). Hence further elucidation of virulence regulatory mechanisms will enable us to better understand *Vibrio*-host interactions and ecology, and to identify targets for the design of novel agents to control disease caused by vibrios.

### Horizontal Gene Transfer, Genome Plasticity, and Chromosome Partitioning

Evolution of *Vibrio* species is often driven by mobile genetic elements via horizontal gene transfer (HGT). However, very little is known about HGT in environmental *Vibrio* isolates infecting marine organisms. In 75 marine *Vibrio* spp. isolated from the broad-nosed pipefish, *Syngnathus typhle*, associated prophages were characterized and the virulence of strains carrying different prophages was then assessed by comparing the relative expression of 44 immune genes during controlled infection experiments on juvenile pipefish. Preliminary results suggest that virulence is significantly influenced by the associated prophages, further supporting a role for bacteriophages in manipulating the virulence of environmental *Vibrio* isolates (C. Wendling unpublished data).

Virulence of *V. vulnificus* and *P. damselae* subsp. *damselae* in fish is determined by transferable plasmids (pVvbt2 in *V. vulnificus* and pPHDD1 in *P. damselae*). pVvbt2 contains two highly conserved virulence genes involved in serum resistance (*vep07*) and the ability to grow from eel transferrin (*vep20*) (Pajuelo et al., 2015). Interestingly, pPHDD1 also contains *vep07* and *vep20* homologs suggesting that both genes are involved in resistance to fish innate immunity. pVvbt2 also encodes RtxA1_3_, a toxin belonging to MARTX (multifunctional, autoprocessive, repeat in toxin) family. RtxA1_3_ is considered a host-non-specific virulence factor because it is involved in resistance to phagocytosis by murine and human phagocytes as well as in eel death (Lee et al., 2013). The other virulence plasmid, pPHDD1, encodes phospholipase-D damselysin (Dly) and the pore-forming toxin HlyA_*pl*_ (Rivas et al., 2011). A second HlyA (HlyA_*ch*_) is encoded in chromosome I (Rivas et al., 2013a, 2014) and the three toxins contribute to hemolysis and virulence, and are secreted by a type-two secretion system (Rivas et al., 2015). While the two HlyA hemolysis produce an additive effect, Dly and any of the two HlyA interact in a synergistic manner, being responsible for maximal virulence for fish and for mice (Rivas et al., 2013a). Due to their host range and their duality as pathogens for both poikilotherm and homeotherm animals, *P. damselae* and *V. vulnificus* constitute valuable biological models to study the role of mobile genetic elements in the rise of novel pathogenic strategies.

Vibrios contain large chromosomal integrons (Cambray et al., 2010) and belong to the group of naturally competent bacteria, which allows them to absorb free DNA from their surrounding environment and recombine it into their genome (Seitz and Blokesch, 2013a). For *V. cholerae*, entry into competence is tightly regulated and requires growth to high cell densities on chitinous surfaces (Meibom et al., 2005; Lo Scrudato and Blokesch, 2012, 2013). Uptake of external DNA is accomplished by a sophisticated DNA-uptake machinery (Seitz and Blokesch, 2013b, 2014; Seitz et al., 2014). As the competence regulon also encompasses the type VI secretion system-encoding gene clusters, HGT is enhanced through deliberate killing of neighboring non-sibling cells followed by the transfer of their DNA (Borgeaud et al., 2015).

The presence of two chromosomes is another characteristic feature of vibrios. While distinctive localization patterns have been described for the two chromosomes, the selective advantages brought by this bipartite architecture are still under debate (Val et al., 2012, 2014). Replication of both chromosomes is tightly coupled so that replication termination is synchronized (Rasmussen et al., 2007). Moreover, the chromosomal position of genes determines the relative copy number during growth thereby impacting the bacteriums physiology (Soler-Bistue et al., 2015). Notably, mechanistic aspects of chromosome organization, architecture, and cell cycle-dependent dynamics are only starting to be deciphered (Yamaichi et al., 2012; Demarre et al., 2014). The elucidation of the mechanisms that coordinate the interplay between chromosomes, accessory replicons, mobile DNA and HGT mechanisms is essential to better apprehend the evolution and niche adaptation of *Vibrio* species.

### Adaptation of Pathogenic Vibrios to Intracellular Life

The pathogenic *V. tasmaniensis* strain LGP32, a member of the *V. splendidus* clade (Gay et al., 2004) was found to be a facultative intracellular pathogen of oyster immune cells called hemocytes (Duperthuy et al., 2011). This is a rare example of *Vibrio* adapted to intracellular life. The virulence of LGP32 in oysters correlated with the ability to enter hemocytes (Duperthuy et al., 2010, 2011). Both cellular invasion and pathogenicity depend on the major outer membrane protein OmpU, which serves as an adhesin to invade host cells. Once inside the phagosome, LGP32 releases outer membrane vesicles (OMVs) that protect the organism against antimicrobial peptides and act as vehicles for the delivery of virulence factors (Destoumieux-Garzón et al., 2014; Vanhove et al., 2015). Moreover, entry into hemocytes and intracellular survival of LGP32 are required for expression of LGP32 cytotoxicity toward hemocytes. This capacity to survive intracellularly relies on potent antioxidant and copper tolerance responses, both of which are highly induced in the hostile environment of the phagosome.

Small regulatory RNAs have been shown to play important roles in regulating virulence gene expression in response to conditions encountered in the host. sRNAs present in multicopies such as Qrrs and CsrBs were found in several *Vibrio* spp. to mediate QS regulation of virulence gene expression (Nguyen and Jacq, 2014). One peculiarity of the *Splendidus* clade seems to be the presence of four highly expressed copies of the CsrB sRNAs in their genome, instead of 2–3 found in other vibrios (Lenz et al., 2005; Toffano-Nioche et al., 2012). CsrB sRNAs are highly transcribed inside oyster hemocytes suggesting a role in adaptation to the intracellular environment (Vanhove et al., submitted). The landscape and phylogeny of putative sRNAs encoded by LGP32 demonstrate rapid vertical evolution, with a vast majority of sRNAs being species/strain specific, and only a small number (28/250) conserved in all *Vibrio* sequenced so far (Toffano-Nioche et al., 2012). Thus, sRNAs contribute to a high diversity between species and provide opportunities for adaptation/colonization of new hosts and virulence emergence, a question that will be tackled by comparative functional studies of conserved *Vibrio* sRNAs.

### Model Systems to Study Pathogenicity Mechanisms and *Vibrio*-host Interactions

Microbiologists are increasingly aware that how organisms behave *in situ* in the “real world” might be distinct from those that occur in laboratory monocultures grown under tightly controlled conditions (Smith, 2000). Thus, model systems, which replicate at least part of the natural processes of infection, are needed in order to examine the relevance and biological impact of *in vitro* findings. *In vivo* models that reproduce the main clinical and pathological signs of disease seen following the consumption of contaminated food or water, are available for toxigenic and non-toxigenic *V. cholerae*, and for *V. parahaemolyticus* (Ritchie et al., 2010, 2012; Shin et al., 2011). In these studies, a combination of microbiological, histological and genetic analysis was used to identify key virulence factors and the pathologic mechanisms associated with the respective strains (e.g., see Zhou et al., 2013, 2014). However, a growing number of *Vibrio*-associated illnesses are associated with a diverse group of strains, some of which lack known virulence factors (Garcia et al., 2009; Jones et al., 2012; Ottaviani et al., 2012). A future challenge will be to examine the pathogenesis of these strains and identify additional virulence markers, which should be used to improve risk assessment tools targeted to the different pathogens. Furthermore, a growing number of human *Vibrio* infections in Europe were not food-borne, but instead associated with the ability of non-O1-non-O139 *V. cholerae*, *V. parahaemolyticus*, or *V. vulnificus* to cause septicemia via wound infections (Table 1). Models to examine this aspect of their pathogenicity are currently lacking and should become a high priority given the poor prognosis of individuals acquiring this type of infection.

Next to models for human pathogens there is also an increasing need for aquatic animal models. However, studies aimed at investigating the pathogenicity mechanisms in aquatic hosts are often confounded by the presence of the natural microbiota (which usually contains *Vibrio* spp.). Gnotobiotic animals provide researchers with a means to examine host-microbe interactions without interference or influence from unknown microbiota (Gordon and Pesti, 1971). A model based on the use of gnotobiotic 1-day old larvae of brine shrimp (*Artemia franciscana*) has been recently developed to study *V. campbellii*, *V. harveyi*, or *V. anguillarum* pathogenesis (Defoirdt et al., 2005). An alternative model system for *V. anguillarum* involves the use of gnotobiotic European sea bass (*Dicentrarchus labrax*) larvae, where survival is monitored over 1 week (Li et al., 2014a). Finally, specific-pathogen-free (SPF) juveniles of *C. gigas* (Petton et al., 2013, 2015) have been developed to investigate the diversity and dynamics of microbial populations in an oceanic environment during disease. When combined with methods to monitor gene expression and activity of vibrios during infection (e.g., Ruwandeepika et al., 2011; Defoirdt and Sorgeloos, 2012), a better understanding of the infection process(es) will emerge.

## Conclusion

**FIGURE 1:**
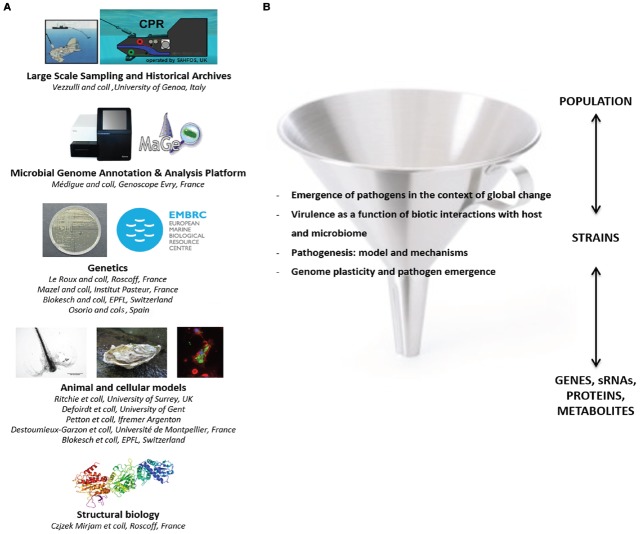
**Perspectives of the European ***Vibrio*** network. (A)** Sharing of common tools and databases. Large-scale sampling for *Vibrio* collection in the environment and retrospective analysis of *Vibrio* populations could be improved using the continuous plankton recorder (CPR) technology and the historical CPR archive. An “encyclopedia of *Vibrio* genome sequences” could be developed by the Genoscope (Evry, France) allowing access for the community to the Microbial genome annotation and analysis platform (MAGE). A genetic resource center, initially created under the scope of EMBRC France, could be improved thanks to other teams performing genetic development. Several teams developing *in vivo* and *in vitro* models to investigate host-*Vibrio* interactions as well as structural biology were also identified. Training sessions (such as summer schools) in the field of bioinformatics or microbial genetics could be organized. **(B)** Collaborations addressing specific questions have already been stimulated by the workshop.

This workshop clearly demonstrated the importance of vibrios to our understanding of emergent diseases in marine and inland aquatic ecosystems as well as their potential impact on society. The rising frequency of disease events not only affects humans directly but also indirectly by reducing food security and ecosystem health. The synergistic investigation of mechanistic and ecological processes contributing to disease is therefore paramount for our understanding of the larger scale consequences of changing *Vibrio* populations. A better understanding of *Vibrio* ecology is pivotal for the development of prevention and mitigation strategies. In addition, the mechanistic knowledge of virulence regulatory mechanisms could ultimately be used to inhibit disease. However, these tasks are complicated by the high diversity present within *Vibrio* populations, and the fact that biotic interactions within and between microbial communities, modify disease expression on different levels. Therefore, we have to consider *Vibrio* disease as an emergent, multi-faceted phenomenon that will require experimental model systems covering molecules to whole organisms. Expertise for most of these crucial challenges already exists and became united at the workshop under the European umbrella of *Vibrio* research thereby fostering a more productive combination of basic and applied research in the future (Figure 1).

### Conflict of Interest Statement

The authors declare that the research was conducted in the absence of any commercial or financial relationships that could be construed as a potential conflict of interest.
